# Masking the Perceived Astringency of Proanthocyanidins in Beverages Using Oxidized Starch Hydrogel Microencapsulation

**DOI:** 10.3390/foods9060756

**Published:** 2020-06-08

**Authors:** Xiaodan Zhao, Yingchao Ai, Yulin Hu, Yongtao Wang, Liang Zhao, Dong Yang, Fang Chen, Xiaomeng Wu, Yuan Li, Xiaojun Liao

**Affiliations:** 1 College of Food Science and Nutritional Engineering, China Agricultural University, Beijing 100083, China; 18810153313@163.com (X.Z.); 2016308010117@cau.edu.cn (Y.A.); hyl19950425@outlook.com (Y.H.); wangyongtao102@163.com (Y.W.); zhaoliang1987@cau.edu.cn (L.Z.); dyang@cau.edu.cn (D.Y.); chenfangch@sina.com (F.C.); yuanli@cau.edu.cn (Y.L.); liaoxjun@cau.edu.cn (X.L.); 2 National Engineering Research Centre for Fruit and Vegetable Processing, Beijing Key Laboratory for Food Nonthermal Processing, China Agricultural University, Beijing 100083, China; 3 Key Lab of Fruit and Vegetable Processing, Ministry of Agriculture and Rural Affairs, China Agricultural University, Beijing 100083, China; 4 Beijing Advanced Innovation Centre for Food Nutrition and Human Health, China Agricultural University, Beijing 100083, China

**Keywords:** astringency, proanthocyanidin, oxidized starch hydrogel, salivary protein precipitation, half-tongue sensory evaluation

## Abstract

Proanthocyanidins (PAs) are responsible for several health benefits of many fruits, but they could cause a generally disliked sensation of astringency. Traditional deastringency methods remove bioactive ingredients, resulting in the loss of valuable nutrients and associated health benefits. This work aimed to microencapsulate PAs from grape seeds using oxidized starch hydrogel (OSH) and mask its perceived astringency in beverages while maintaining its bioavailability. The maximum PA uptake capabilities of OSH, as well as the binding site and primary binding force between these two components, were determined. The resulting PA-OSH complex was stable under in vitro digestion, with only 1.6% of PA being released in the salivary digestion, and it has an intestine-specific release property. The reaction of PA with α-amylase in artificial saliva was substantially reduced by OSH microencapsulation, leading to 41.5% less precipitation of the salivary proteins. The sensory evaluation results showed that the microencapsulation was able to mask the astringency of PA-fortified water, as the perceived threshold of astringency increased by 3.85 times. These results proved that OSH could be used as a novel food additive to reduce the astringency of beverage products due to its hydrogel properties and ability to encapsulate phenolic compounds.

## 1. Introduction

Fruits are essential food sources and play a key role in human health; almost 640 million tons of fruits were gathered throughout the world in 2011 [1]. Many fruits contain a high level of polyphenols, including phenolic acids, flavonoids, stilbenes, and lignans, which have multiple health benefits, such as antioxidant, anti-inflammatory, anti-cancer, and anti-obesity [2] properties. Besides this, these compounds can also promote cardiovascular health and improve bone quality [3]. However, some polyphenols, especially condensed tannins, can elicit an astringent taste, and this sensation becomes more intense in liquid food products with high polyphenol contents, such as fruit wine, tea, and juice [4]. Although it is considered a unique and characteristic sensory attribute of red wines [5], the astringent taste is generally considered as a disliked sensation in other food products. It has been reported to be negatively associated with the preference and acceptability of polyphenol-rich products [6], influencing the consumption of these non-alcoholic beverages.

Astringency has been recognized as a complex oral sensation involving the drying, roughing, and puckering of the mouth epithelia [7]. Many mechanisms of astringency have been proposed, including the loss of lubrication, the inactivation of neuroepithelial taste receptor cells (TRCs), and the interaction of polyphenols with salivary proteins [8]. Among them, the most established mechanism involves the interaction of astringency-causing polyphenols and certain salivary proteins, such as proline-rich proteins and α-amylase [9]. This interaction induces the aggregation and precipitation of the salivary proteins, eliciting the sensation of dryness and the astringency feeling.

Astringency can be measured by sensory evaluation tests and equipment such as the electronic tongue for rapid prediction. A sensory evaluation is also performed to measure astringency with panel tastings, in which a points scale is always used in order to judge the stage of astringency [10]. In other sensory tests, the intensity of astringency is evaluated by calculating the change in the astringency of samples between two consecutive sips [11]. The half tongue test is a recently developed quantitative method to evaluate the astringent taste [12]. Panelists were asked to identify which half of their tongue had the astringency-causing agents when the testing and the control samples were applied to each half of the tongue. This method provides quantitative data on the perceived astringency threshold of samples.

Many treatments have been adopted to reduce or even eliminate the undesirable taste of astringency in food products. Current deastringency methods widely used in the food and beverage industry usually involve chemical precipitation, using flocculants such as proteins [13], or physical adsorption, using active carbons [14]. These methods are also referred to as the fining process in winemaking [15]. Despite the different mechanisms and additives used, these methods essentially achieve a reduction in astringency by removing polyphenols and tannins from food products. These polyphenolic compounds are often associated with the health benefit of the food product, so removing them will lead to a loss of nutrition and product value. An effective deastringency method without sacrificing the nutrient value nor changing the flavor and physical property of the food is still yet to be developed. The intense astringent taste is most noticeable in wines and juices made from fruits such as aronia berry (chokeberry), sea buckthorn, and red currant [16]. In order to reduce the astringency of these beverages, the added deastringency agents need to be water-soluble so that they will not introduce unnecessary precipitation or the separation of the products. Hence, a water-soluble colloid that can encapsulate the astringency-causing tannins in fruit beverages is ideal for such applications.

With the benefits of being low cost and non-allergic, starch-based colloids have received increasing interest for microencapsulation applications [17]. In order to improve the encapsulation ability of the starch and introduce extra features, such as the hydrophobicity and viscosity, starches have been modified chemically and enzymatically. Previous studies have reported that the octenyl succinic anhydride (OSA) modified starch showed an increased hydrophobicity for better microencapsulation and controlled release [18]. The ferulic acid esterified starch presented increased water-holding capacity, reduced viscosity, and much less retrogradation during low-temperature storage than native starch [19]. Enzymatically modified cassava starches by fungal lipase are widely used in the controlled release of drugs and bioactive compounds due to their superior embedding properties [20].

Among all these starch modification methods, the oxidation mediated by 2,2,6,6-tetramethyl-1-piperidinyloxy (TEMPO) is a highly efficient and controllable approach to prepare starch hydrogel [21]. Compared with natural starch, TEMPO-oxidized starch hydrogel (OSH) has good fluidity and high viscosity, and it was proven to have negligible toxicity in several cell lines and ICR mice [22]. Therefore, it has been widely accepted as a drug delivery agent due to its excellent biodegradability and compatibility. Most importantly, TEMPO-oxidized potato starch is water-soluble and can form a negatively charged colloidal solution in an acidic environment [23]. Therefore, it has been used to microencapsulate and deliver positively charged compounds, such as lysozymes nanoparticles, in liquid [24]. The polysaccharides could bind with polyphenols through either electrostatic interaction [25] or hydrogen bonding [26]. Therefore, in theory the OSH could also microencapsulate positively charged polyphenols, which are responsible for astringency in food. The solubility and microencapsulation ability of OSH would make it an ideal food additive for liquid food applications.

Here, we propose a new deastringency method in beverages using OSH to microencapsulate astringency-causing compounds. Hypothetically, such microencapsulation would inhibit the interaction of polyphenols and salivary proteins and eventually reduce astringency in the oral cavity. The microencapsulated polyphenols can then be released and utilized in the gastrointestinal tract after the digestion of OSH in order to achieve the purpose of masking the astringent taste while retaining the bioavailability. Since proanthocyanidin (PA) is the second most abundant natural polyphenolic compound in fruits and responsible for the astringent sensation of many fruits [27], we use PA to test the feasibility of the method. In this paper, the maximum adsorption and distribution of PA by OSH are determined, and the interaction between PA and OSH is studied. The release of PA is analyzed in vitro using simulated salivary, gastric, and intestinal fluids. The effect of OSH on reducing the salivary protein precipitation caused by PA is investigated. The half-tongue sensory evaluation is conducted to demonstrate OSH’s capability to decrease the perceived astringency of PA-fortified water beverages.

## 2. Materials and Methods

### 2.1. Materials

Food grade proanthocyanidins (PAs) extracted from grape seed with a >95% purity were purchased from Beijing Solarbio Science and Technology Co., Ltd. (Beijing, China). Native potato starch was provided by AVEBE (Veendam, Netherlands). The oxidation catalyst 2,2,6,6-tetramethyl-1-piperidinyloxy (TEMPO) was purchased from Merck KGaA (Darmstadt, Germany). The sodium trimetaphosphate (STMP), gastric mucin, α-amylase, bile, and duodenal juices were supplied by Sigma-Aldrich (St.Louis, MO, USA). Artificial saliva was purchased from Shanghai Yuanye Biotechnology Co., Ltd. (Shanghai, China). The α-amylase labeled with fluorescein isothiocyanate (FITC) was purchased from Beijing Anbiqi Biotechnology Co., Ltd. (Beijing, China). Among the chemicals used, citric acid and sodium citrate were of food-grade, and all the other chemicals used were of analytical grade.

### 2.2. Preparation of OSH

Oxidized starch hydrogel (OSH) was prepared based on the previously reported method [28]. The experimental conditions were optimized for PA adsorption in the preliminary study. Briefly, the starch was selectively oxidized at the 6-position by TEMPO-mediated oxidation to achieve a polyglucuronate with a >95% selectivity, resulting in starch polymers of 50% degree of oxidation (DO). Subsequently, 20 g of the oxidized starch polymer was dissolved in 95 mL of distilled water at room temperature and then cross-linked by STMP with 2.0 M sodium hydroxide at 40 °C for 10 min without stirring. The gel was heated in a conventional oven at 40 °C for 1 h to form the OSH. The OSH was kept in the refrigerator at 4 °C overnight for further cross-linking and then was passed through a sieve (1 mm) covered with a nylon cloth of 200 mesh (mesh size 0.074 mm). The OSH particles were dried in the oven at 40 °C overnight. Finally, the dried powder was again passed through the colloidal mill to obtain small and homogenous particles. The particle size of the resulting OSH generally ranged from 10 to 20 μm.

### 2.3. PA Adsorption of the OSH

First, the PAs were measured and dissolved in a citric acid/sodium citrate buffer (pH at 3) with 0.45 μm filters to prepare the PA solution at 0.8–4.0 g/L. The OSH solution was prepared using the same citric acid/sodium citrate buffer to reach 1 g/L. The PA solution was added to the OSH solution with gentle stirring for 2 h to allow the reaction to take place. Subsequently, the PA-OSH solution was centrifuged at 10,000 rpm and 4 °C for 10 min, and the concentration of PA in the supernatant was determined using UV spectrophotometry with a modified vanillin-sulfuric acid method [29]. Briefly, 0.5 mL of PA-containing solution was mixed with 2.5 mL of 30 g/L vanillin in methanol and 2.5 mL of sulfuric acid in methanol (30% *v/v*) to react in the dark for 20 min at 37 °C. The absorbance was read at 500 nm using a UV-vis spectrophotometry (UV-1800, Shimadzu, Japan), and PA concentration was calculated using a pre-established standard curve.

The PA uptake capacity of OSH (Γ) was defined as the amount of PA absorbed per weight of dry OSH, and it was calculated as follows [28]:

(1) $$\Gamma =(C_{\mathrm{PA}\text{-}\mathrm{total}}-C_{\mathrm{PA}\text{-}\sup})V_{\mathrm{PA}}/m_{\mathrm{OSH}},$$

where Γ (mg of PA/g of OSH) is the PA uptake capacity of OSH, *C*_PA-total_ (g/L) is the PA concentration in the solution before adding the OSH, *C*_PA-sup_ (g/L) is the PA concentration in the supernatant after the reaction, *V*_PA_ (L) is the volume of the PA solution, and *m*_OSH_ (g) is the mass of the dry OSH added.

### 2.4. Zeta-Potential Assessment and CLSM Characterization

Samples of the PA-OSH mixtures used for the ζ-potential assessment and obtaining confocal laser scanning microscopic (CLSM) images were prepared according to the aforementioned procedure. The assessment of the ζ-potential was conducted using a Zen 3700 Zetasizer (Malvern, UK) according to a previous method with slight modifications [30]. The CLSM images and fluorescence intensity of the PA (a natural fluorescent dye), PA-OSH mixtures, and FITC-labeled α-amylase was were obtained using a Nikon A1Rsi microscope (Nikon Inc., Tokyo, Japan). The excitation and emission wavelengths were set at 488 and 525 nm, respectively, for Channel 1; the excitation and emission wavelengths were set at 561 and 595 nm, respectively, for Channel 2.

### 2.5. ITC Analysis

Isothermal titration calorimetry (ITC) was employed with a Nano ITC instrument (TA Instruments Ltd., Crawley, UK) according to a previously reported method with slight modifications [31]. The reference cell was filled with water, whereas the reaction cell was filled with 300 μL of the OSH solution. The 40 mM PA solution was injected into the reaction cell at intervals of 200 s with 200 rpm stirring. Each 2.5 μL titrant of 20 injections was sequentially delivered into the reaction cell. The heat profile was fitted with the multiple site model to obtain the thermodynamic parameters. The Gibbs free energies and entropic contributions were calculated using the equations ΔG = *RT* ln *K_d_* and ΔG = ΔH − TΔS, respectively [31].

### 2.6. In Vitro Digestion

For each in vitro incubation, aliquots of PA-OSH solution (PA = 2.8 g/L, OSH = 1 g/L) were digested, while the 2.8 g/L of pure PA solution was used as control. The procedure was adopted with modifications [32]. For salivary digestion, 2.5 mL of the simulated saliva fluid containing mucin (1 g/L) and α-amylase (2 g/L) was added to an aliquot of 10 mL of the PA or PA-OSH solutions, and the pH was quickly adjusted to 6.8 using NaOH (1M). The mixture was incubated at 37 °C in a shaking bath for 10 min, and the PA concentration in the solution was measured using the aforementioned vanillin-sulfuric acid method every two minutes. At the end of the salivary digestion phase, 5 mL of simulated gastric fluid with mucin (6 g/L) and pepsin (5 g/L) was added to the mixture, and the pH was adjusted to 2.8 using 1 M HCl. It was incubated for 50 min at 37 °C in a shaking bath, while the PA concentration was measured every 10 min using the same method mentioned above. The intestinal digestion was initiated by adjusting the pH to 7.0 using NaOH (1 M). After adding 2.5 mL of bile juice (60 g/L bile) and 5 mL of duodenal juice containing pancreatin (18 g/L) and lipase (3 g/L), the mixture was incubated at 37 °C for 180 min. The amount of PA released from the complex into the solution was measured at 70, 80, 90, 100, 110, 120, and 240 min from the beginning of the in vitro digestion process using the same method. Since the PA would be digested after being released from the PA-OSH, the pure PA was used to measure the percentage of PA degradation in the solution. The amount of released PA was adjusted to reflect such degradation as follows:

(2) $$\mathrm{Und}\%=1-\mathrm{Deg}\%=(C_{\mathrm{PA}\text{-}\mathrm{control}}/C_{\mathrm{PA}\text{-}\mathrm{ini}})\times 100\%,$$

(3) $$\begin{array}{ll} \mathrm{Adjusted}\;\mathrm{Release}\;(\%) & =(C_{\mathrm{PA}\text{-}\mathrm{release}}/C_{\mathrm{PA}\text{-}\mathrm{OSH}\text{-}\mathrm{ini}})\times 100\%\\ & =(C_{\mathrm{PA}\text{-}\mathrm{test}}/\mathrm{Und}\%/C_{\mathrm{PA}\text{-}\mathrm{OSH}})\times 100\%\\ & =(C_{\mathrm{PA}\text{-}\mathrm{test}}/C_{\mathrm{PA}\text{-}\mathrm{control}})/(C_{\mathrm{PA}\text{-}\mathrm{OSH}\text{-}\mathrm{ini}}/C_{\mathrm{PA}\text{-}\mathrm{ini}})\times 100\% \end{array},$$

where Und% is the percentage of PA not degraded during digestion, Deg% is the percentage of PA degraded in the solution, and Adjusted Release is the adjusted release of PA from PA-OSH complex. *C*_PA-test_ and *C*_PA-control_ are the concentrations of PA in the solution of PA-OSH samples and pure PA samples, respectively, measured at the same time during digestion. *C*_PA-OSH-ini_ and *C*_PA-ini_ are the concentrations of PA in the PA-OSH samples and pure PA samples before digestion, respectively.

### 2.7. SDS-PAGE and SPI Analysis

The enzyme-added artificial saliva (EAAS) was prepared by adding 500 mg of α-amylase (1000–3000 units/mg, from human saliva) and 500 mg of mucin into 100 mL of commercially available enzyme-free artificial saliva, based on previously reported methods with slight modifications [8,33]. It was then mixed with PAs at 0.25 to 1.5 g/L or the saturated PA-OSH complex, followed by incubation at 37 °C for 5 min. The mixture was centrifuged at 10,000× *g* for 10 min at 4 °C to remove insoluble impurities, and the supernatant was used for electrophoresis.

The sodium dodecyl sulfate-polyacrylamide gel electrophoresis (SDS-PAGE) was performed on a Bio-Rad Mini-PROTEAN Cell (Bio-Rad Laboratories Ltd., Philadelphia, PA, USA) using a power supply set at 90 V/gel for the stacking gel and 120 V/gel for the resolving gel. The samples were mixed with an equal volume of 2x electrophoresis sample buffer and heated at 100 °C for 10 min, then were analyzed by SDS-PAGE. After the electrophoresis, the gel was stained in the Coomassie Brilliant Blue R250 staining solution and heated in a microwave oven for 50 s on high heat and shaken for one hour on a shaker. The gel was then decolorized with a high methanol decolorizing solution overnight until the background color of the gel was nearly colorless.

Since we are more interested in the relative change than in the exact amount of salivary protein content in the samples, the salivary precipitation index (SPI) was determined according to a previously reported method [34]. We defined the SPI as the percentage reduction in the density of protein bands in the test group (PA or PA-OSH added EAAS) compared to that in the control group (pure EAAS). The SDS-PAGE gel was scanned by a CanoScan Lide 120 scanner (Canon USA Inc., Melville, NY, USA). The apparent molecular weights of the bands were calculated from the linear regression equation of log molecular weight against mobility, by comparison with the migration rates of broad range protein standards. The density of each band was determined using Image J software, and the SPIs of the testing samples were calculated as follows,

(4) $${\mathrm{SPI}(\%)=(\mathrm{Density}}_{\mathrm{control}}-{\mathrm{Density}}_{\mathrm{test}}{)/\mathrm{Density}}_{\mathrm{control}}\times 100\%,$$

where Density_test_ is the protein band density of the testing sample and Density_control_ is the protein band density of the control sample.

### 2.8. Half-Tongue Sensory Evaluation

The human astringency recognition threshold (HART)—the lowest concentration at which the astringency of a compound is detectable—of the sample was evaluated using a half-tongue sensory evaluation method adopted from previous literature with slight modifications [35]. The sensory tasting panelists between the ages of 20 and 35 had received proper training before the final sensory evaluation. In the training sessions, 0.001% gallic acid in citrate buffer (pH at 3) was used in the half-tongue test for the panelist to become familiar with astringency, roughness, and dry mouth feelings, as well as the test procedure. The half-tongue test in the training session was also used to determine the panelists’ HART of gallic acid. In the end, 26 panelists with similar HARTs were selected into the final sensory tasting panel. All the sensory analyses were performed in a sensory panel room at 15–25 °C. No ethical approval was required for this study.

In the half-tongue test, the panelists were presented with PA or PA-OSH-fortified water in order of increasing concentration using the sip-and-spit method. The fortified water samples were prepared with drinking water, 0.1 M food-grade citric acid and sodium citrate, and PA or PA-OSH of various concentrations. For each panelist, his/her tongue was divided into two sections, the left and right. Starting from the lowest concentration, an aliquot (~0.2 mL) of the testing solution (gallic acid for the training session and PA/PA-OSH-fortified water for the sensory test) was applied on one section of the tongue, whereas an aliquot (~0.2 mL) of the blank control was applied to the other section at the same time as the control. The panelists were then asked to stretch their tongues back and forth for 15 s and then to identify which section of the tongue they felt the astringent taste on. To avoid fatigue, the panelists were asked to rinse with pure water and rest for 10 min before proceeding to the next set of samples. The starting concentration of PA was set at two steps below the threshold concentration determined in a preliminary taste experiment. If the panelists identified the incorrect section, a beverage containing a higher concentration PA/PA-OSH was then used. If the panelists correctly identified the section with testing solutions, the same concentration was presented for two more times as a proof for the correctness of the data. The geometric mean of the last and the second last concentration was calculated and taken as the individual HART of the testing solutions.

### 2.9. Statistical Analysis

All the experiments were carried out at least in triplicate and reported as the mean ± standard error. All the statistical analyses were performed using a one-way analysis of variance (ANOVA) with Duncan’s test by IBM SPSS Statistics 21 (SPSS Inc., Chicago, IL, USA). The sensory evaluation test results were also analyzed using ANOVA with the data obtained from the 26 panelists. All the illustration drawings were constructed using OriginPro^®^ 9.2 (OriginLab Co., Northampton, MA, USA) and Adobe^®^ Photoshop^®^ CC 2018 (Adobe Systems, San Jose, CA, USA).

## 3. Results and Discussion

### 3.1. The Absorption of PA by OSH and Distribution

In order to evaluate the PA microencapsulation ability of OSH, it is critical to analyze the adsorption isotherm of PA by OSH in the first place. The OSH was optimized to achieve the highest PA microencapsulation in the preliminary studies. The PA uptake capacity of the optimized OSH (Γ) as a function of the PA concentration was plotted in the supplementary data (Figure S1), while the OSH concentration was kept constant at 1 g/L. The PA uptake capacity of OSH (Γ) gradually increased with the increasing PA concentration and reached a plateau at 2.8 g/L PA concentration. The maximum adsorption is at 850 mg of PA/g of OSH. The previous study reported the uptake and release of anthocyanins by OSH [28]. They found that a maximum of 62 mg anthocyanins can be absorbed per gram of OSH, which was lower than the PA absorption we reported here, possibly due to PA’s more massive molecular weight. In addition, it has been reported that charged hydrogel with a porous structure had a higher PA absorption capacity than natural starch [25], possibly due to the increased accommodation capacity of the oxidized starch polymer network [36].

To further understand the interaction between PA and OSH, the ζ-potential of the PA-OSH complex was measured at different PA concentrations in the supplementary material (Figure S2). The ζ-potential is widely considered as an important indicator of the strength of electrostatic repulsion or attraction between particles [37]. A higher absolute value of ζ-potential often indicates smaller molecular or dispersed particles and a more stable colloidal system [38]. Conversely, a lower absolute value of ζ-potential suggests that the system is more prone to coagulation or agglomeration. In Figure S2, the ζ-potential of the OSH itself was −7.47 mV, whereas the ζ-potential of the PA-OSH complex became more negative (increase in absolute value) with the increase in PA concentration from 0 to 2.0 g/L.

The negatively charged complex was gradually compensated for by the positively charged PA, leading to a more stable system. When the concentration of PAs exceeded 2.8 g/L, the ζ-potential of the PA-OSH complex remained almost constant at −11 mV, indicating that the absorption had reached saturation. These results confirmed that the OSH could interact with the PA at least partially via electrostatic interaction, and the resulting PA-OSH hydrocolloids were stabilized by charge repulsion.

**Figure 1:**
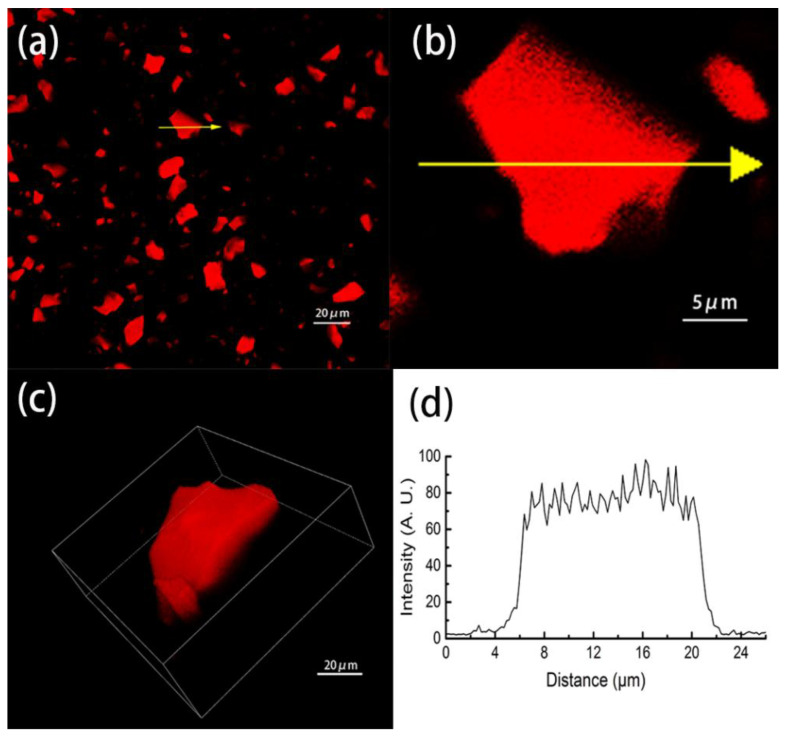
The confocal laser scanning microscopic (CLSM) images of the saturated proanthocyanidin (PA)-oxidized starch hydrogel (OSH) complexes. (**a**) The overall image of the PA-OSH complexes. A single PA-containing OSH particle is marked with a yellow arrow. (**b**) The enlarged image of the yellow arrow-marked OSH. (**c**) Three-dimensional model of the yellow arrow-marked OSH. (**d**) The fluorescence intensity of the marked OSH particle along the yellow arrow in Figure 1b.

Owing to the three-dimensional porous structure of OSH [39], theoretically, PAs can simultaneously diffuse into all parts of the hydrogel. In order to observe the distribution of PAs inside the OSH after absorption, the precipitation of the oversaturated PA-OSH complex (PA = 4 g/L, OSH = 1 g/L) after rinsing was examined using confocal laser scanning microscopy (CLSM). As natural fluorescent dyes, PAs appear bright red in CLSM images, and they had a maximum fluorescence intensity at ~390 nm, similar to the previously reported results [40]. In Figure 1a, all the OSH appeared bright red with no visible red dots in the dark background, indicating that the OSH absorbed almost all the PAs. A single PA-OSH particle, yellow-arrow highlighted in Figure 1b, was selected and enlarged in Figure 1b. Moreover, the 3D image of this selected particle was constructed in Figure 1c. The uniform red color was observed across all parts of the hydrogel in Figure 1b,c, suggesting the homogenous distribution of the PA on the surface of the OSH.

In order to further analyze the PA absorption inside the OSH, the fluorescence intensity of this highlighted PA-OSH particle is measured along the yellow arrow in Figure 1b, and the results are presented in Figure 1d. The fluorescence intensity was almost zero from 0 nm to 7 µm, as it represented the portion outside the PA-OSH particle. It suddenly rose to ~80 A.U. at 7 µm, indicating that the scan had reached the surface of the OSH particle. If PAs only accumulated at the surface of OSH, this plot would only have two sharp peaks, corresponding to the two outer surfaces of the OSH, with zero fluorescence intensity in-between. However, the fluorescence intensity between 7 and 20 µm remained at 80 A.U. with limited fluctuation, indicating that there was a uniform distribution of PA within the interior of the OSH.

### 3.2. The Interaction between PA and OSH Studied by ITC

**Figure 2:**
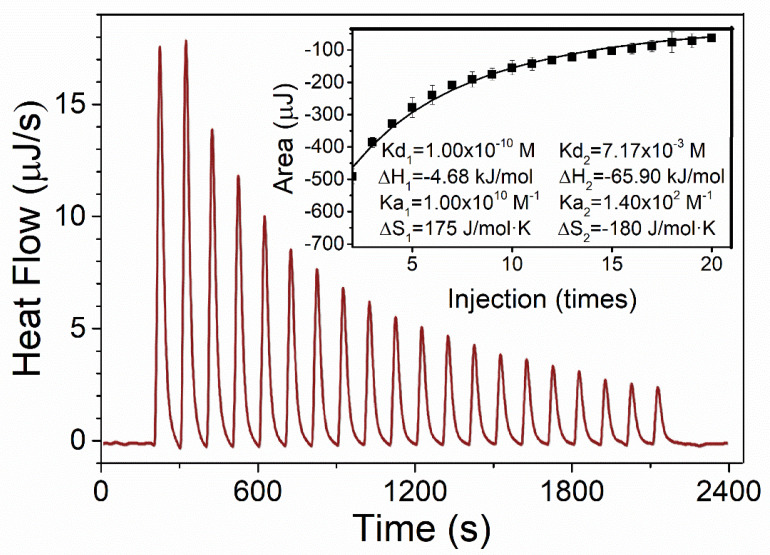
Heat flow vs. time profiles resulting from the injection of 50 μL aliquots of 40 mM PA solution into a 300 μL reaction cell containing 1 g/L of OSH solution. The inserted Figure depicts the binding isotherm; the dots represent the integrated binding heat, and the full line is the fit of a multiple-site binding model to the data.

To further explore the nature of the interaction, isothermal titration calorimetry (ITC) was used to measure the heat change during the process. Figure 2 showed that upon interaction with PAs, the PA-OSH system released heat gradually. According to the Gibbs function and van ’t Hoff equation [41], two sets of enthalpy and entropy changes during this binding process were derived. For the first set, the ΔH_1_ was −4.816 kJ/mol and the ΔS_1_ was 1.751 × 10^2^ J/mol·K. For the second set, the ΔH_2_ was −65.9 kJ/mol, whereas the ΔS_2_ was −1.800 × 10^2^ J/mol·K. These results indicated that the interaction between PA and OSH was dominated by hydrogen bonding, as suggested by the second set of largely negative ΔH and ΔS, with attribution from electrostatic interaction, as suggested by the first set of negative ΔH and positive ΔS. The previous literature reported that electrostatic interaction was the dominant force between OSH and anthocyanins, while hydrogen binding also played an important role [28]. The structural differences between PAs and anthocyanins might be the reason for such discrepancy. The PAs generally have more hydroxyl groups or phenolic groups than anthocyanins, which would form more hydrogen-binding sites with oxygen atoms of the carbohydrate [42].

### 3.3. PA Release under In Vitro Digestion

**Figure 3:**
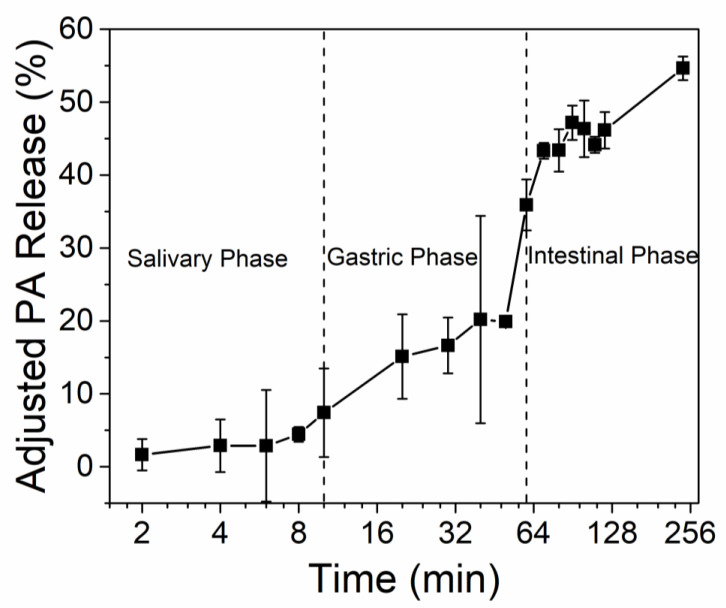
Adjusted PA release (%) from PA-OSH complexes under in vitro digestion. Salivary phase: digestion with simulated salivary fluids (SSF) containing mucin and α-amylase. Gastric phase: digestion with simulated gastric fluid (SGF) with mucin and pepsin. Intestinal phase: digestion with duodenal juice and bile juice.

The OSH is a starch-based polymer that will eventually be enzymatically degraded in the human digestion system. In order for it to act as an astringency-masking agent, the PA-OSH complex has to remain stable in the oral cavity while being able to release PA mainly in the intestine. Therefore, we investigated the release of PA from the PA-OSH complex under in vitro digestion using simulated salivary fluids (SSF), simulated gastric fluids (SGF), and simulated intestinal fluids (SIF) subsequently. The adjusted release of PA from the PA-OSH was plotted as a function of digestion time in Figure 3. In the salivary phase, 1.6% of PA was release in 2 min, and less than 8% was released in 10 min of digestion. Although the average chewing time of each mouthful is usually under 2 min [43], we purposely extended the salivary phase to 10 min to demonstrate the PA-OSH’s stability even with extremely long exposure in the oral cavity. Such excellent stability was due to the α-amylase-resistant property of OSH, mainly resulting from the cross-linking of oxidized starch polymers by STMP. The high degree of oxidation on the starch interfered with its interaction with α-amylase, resulting in increased resistance to the enzyme [44]. The amount of PA released in the salivary phase was also partially due to the PA uptake capacity decrease when the pH increased in the SSF [26]. After salivary digestion, the PA release gradually increased to 19.9% at the end of the gastric digestion phase (50 min), suggesting that limited OSH was digested. It increased substantially when the PA-OSH was subjected to the intestinal conditions and then eventually became 54.6% at 240 min, probably due to the enzymatic digestion of OSH by the pancreatin in the duodenal juice. The pH increase in the intestinal phase could also contribute to the elevated PA release. It has been reported that more anthocyanins were released from OSH at pH 7 than at pH 2 due to the reduced electrostatic reaction [28]. Similar polyphenol release profiles were reported when waxy maize starch nanoparticles were used as a carrier, and up to 80% of the polyphenols were released within 4 h of gastric and intestinal digestion [45]. The intestine-specific release property of OSH would benefit the further adsorption and nutritional utilization of PA in intestinal conditions.

### 3.4. Interaction between PA/PA-OSH and Salivary Proteins

It has been proposed that astringency is mainly due to the complex sensory of polyphenols combined with salivary proteins [8]. The reaction between salivary proteins and PA causes aggregation and precipitation, resulting in an intense astringent sensation. Among all the salivary proteins, α-amylase was found to be most abundant in the precipitates when they were subjected to polyphenols [46], so it was further analyzed as a model salivary protein in this study. We have demonstrated that OSH can absorb PA inside. Therefore, such microencapsulation would impair the opportunity for PA to react with surrounding α-amylase and then reduce the extent of protein precipitation, leading to a subsequent decrease in astringency. In order to test this hypothesis, an SDS–PAGE electrophoresis and densitometry analysis was conducted to evaluate the effect of OSH on PA–protein interaction using α-amylase-added artificial saliva.

**Figure 4:**
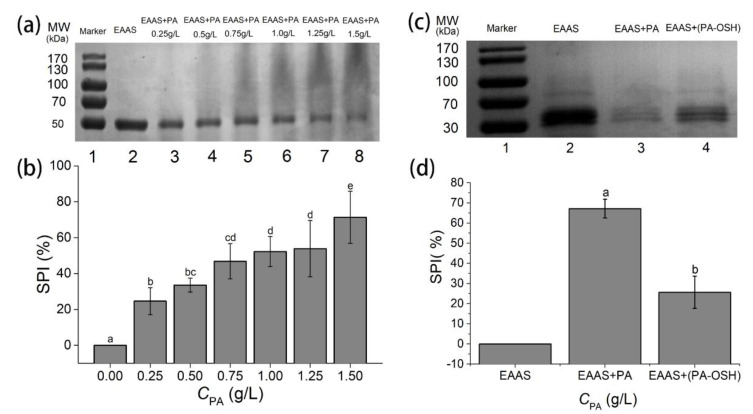
SDS–PAGE and densitometric analysis of enzyme-added artificial saliva (EAAS). (**a**) SDS–PAGE patterns of the EAAS solution reacted with PAs at different concentrations, and (**b**) their salivary precipitation index (SPI). (**c**) SDS–PAGE patterns of EAAS solution reacted with 2 g/L PA solution and saturated PA-OSH complex and (**d**) their SPI. The α-amylase bands are at 54–60 kDa. Columns with different letters are significantly different (*p* < 0.05).

Figure 4a showed the SDS-PAGE patterns of the supernatant in enzyme-added artificial saliva (EAAS) bound with PAs at different concentrations. When it reacted with PA, α-amylase precipitated and then was removed by centrifugation, resulting in a decrease in the abundance of the supernatant. As the amount of PA increased (lanes 3–8), a progressive reduction in α-amylase abundance was observed. To quantify such change, the density of the α-amylase band in each lane in Figure 4a was measured by a densitometric analysis. Using EAAS without any PA as the control, the percentages of density reduction of the α-amylase band in each sample were calculated and plotted as saliva precipitation index (SPI) in Figure 4b [47]. A positive correlation between the PA concentration and the SPI of EAAS samples was observed. This result was consistent with a previous study that showed that tannins significantly precipitated the α-amylase presented, especially in polyphenol-rich products such as red wines [34].

Furthermore, the similar SDS-PAGE experiments were conducted to evaluate the effect of OSH on the interaction between PA and α-amylase in EAAS. As shown in Figure 4c, the EAAS with the PA-OSH complex in lane 4 exhibited a higher α-amylase abundance than the EAAS with PA in lane 3. When OSH was used to absorb and microencapsulate the PA, it substantially reduced the amount of unbound PA in the solution, leading to a decrease in precipitation and a subsequent increase in α-amylase abundance in the supernatant. Similarly, the SPI of each lane was calculated and plotted in Figure 4d. When 1.5 g/L PA was added to EAAS, the SPI was at 67.14% ± 4.62%, while the SPI was significantly lower at 25.65% ± 8.00% (*p* < 0.05) when the same amount of PA was first microencapsulated by 1 g/L of OSH instead. It proved that OSH microencapsulation could effectively prevent the interaction between PA and α-amylase.

**Figure 5:**
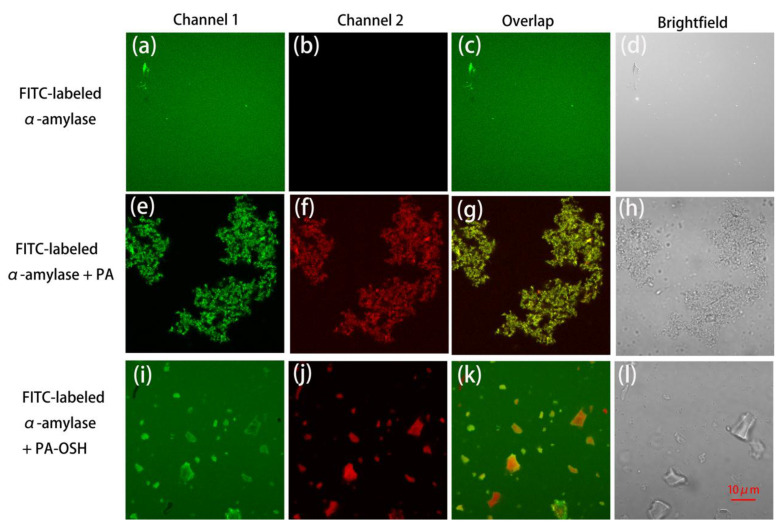
The confocal laser scanning microscopic images of FITC-labeled α-amylases and their reactions with PAs and PA-OSH complexes. (**a**–**d**) The images of FITC-labeled α-amylases. (**e**–**h**) The images of FITC-labeled α-amylases mixing with PA. (**i**–**l**) The images of FITC-labeled α-amylases mixing with PA-OSH. Channel 1: excitation wavelength at 488 nm and emission wavelength at 525 nm. Channel 2: excitation wavelength at 561 nm and emission wavelength at 595 nm. Overlap images are the combined images of Channel 1 and Channel 2.

In order to further visually illustrate the effect of OSH microencapsulation on preventing the aggregation of α-amylase caused by PA, α-amylase was labeled with fluorescein isothiocyanate (FITC), which exhibited green fluorescence with an excitation wavelength at 488 nm and an emission wavelength at 525 nm (Channel 1). Figure 5 showed the images of the FITC-labeled α-amylases and their reaction with PAs and PA-OSH complexes under CLSM. In Figure 5a,c, evenly distributed FITC-labeled α-amylases were observed as the green dots in the Channel 1 image and overlap view. The natural fluorescence of PA would exhibit red color in the Channel 2 images (excitation wavelength at 561 nm and emission wavelength at 595 nm). Figure 5b appeared empty since there was no PA in the system yet. When the PAs were added to the α-amylase solution, their interaction caused the aggregation, and all four views were clouded with clusters of α-amylases-PA aggregates, as shown in Figure 5e–h. The green clusters in Figure 5e represented the FITC-labeled α-amylases, and the red clusters in Figure 5f represented the PAs. The clusters in Figure 5e,f were of identical shapes, which proved that the clusters are indeed caused by the interaction of α-amylases and PA. When OSH encapsulated PAs was added into the α-amylase solution, such aggregation was not observed, as demonstrated in Figure 5i–l. Instead, green dots corresponding to the FITC-labeled α-amylases exhibited a homogenous distribution in Figure 5i,k, which were similar to the ones in Figure 5a,c. Furthermore, the red-colored PA-OSH particles dispersed in the solution, as shown in Figure 5j. These results confirmed our SDS-PAGE results in Figure 4 that OSH microencapsulation could significantly inhibit the precipitation of the salivary proteins by PA, which would lead to a reduction in astringency. Previous studies showed that other carbohydrates had similar protein–PA aggregation prevention capability, and the performance varied depending on their structures [48]—i.e., xanthan gum showed a good inhabitation ability, while acacia gum was not effective against the protein-polyphenol aggregation [42]

### 3.5. Sensory Evaluation for Decreasing the Astringent Threshold by OSH

**Figure 6:**
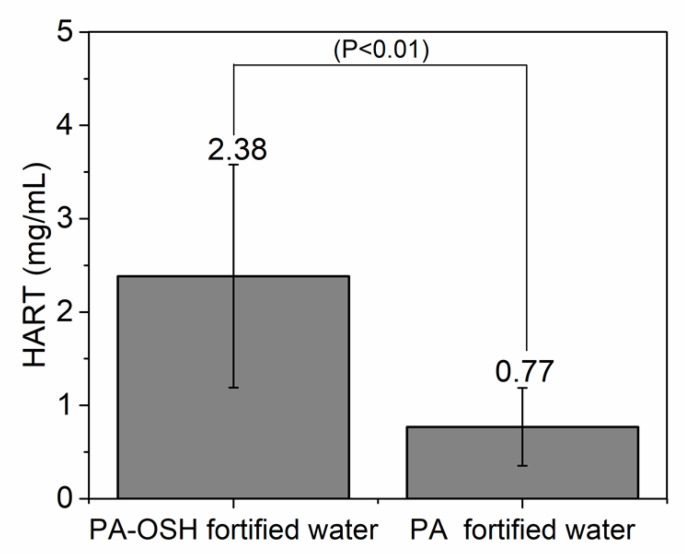
The human astringency recognition thresholds (HART) of the PA and PA-OSH-fortified water determined by the half-tongue sensory evaluation with 26 trained and screened panelists. The level of significance (*p*-value) between the two testing groups is shown in the figure.

In order to demonstrate the effect of OSH microencapsulation in masking the astringent taste of PA in food, the human astringency recognition thresholds (HART) of PA and PA-OSH fortified water samples were determined by utilizing the half-tongue sensory evaluation test with 26 trained and screened panelists. As shown in Figure 6, the HART of PA fortified water was at 0.77 ± 0.23 g/L. When 1 g/L OSH was used for PA microencapsulation, the panelists could only detect the astringent taste in the fortified water at PA concentration of 2.38 ± 1.43 g/L and above, which is 3.85 times higher than the non-encapsulated PA. This suggested that the OSH microencapsulation effectively masked the astringency sensation of PA in the beverage. One may use this microencapsulation technique to produce fortified/enriched beverages with higher PA content without the disliked astringent taste. As we demonstrated in the previous section, the precipitation of α-amylase caused by PA substantially diminished after the OSH microencapsulation (Figure 4). Since α-amylase precipitation is the leading cause of astringency, such reduction in salivary protein precipitation would be the reason for the increased HART of the beverage.

It was reported that fining with gelation and potato protein reduced the astringency intensities of the wines by 31.3% and 31.6%, respectively [15]. In addition, patatin, gelatin, egg albumin, and potassium caseinate were reported to be able to significantly reduce the astringency rating of the Aglianico wines by 20.3% to 35.6% [49]. The degree of astringency reduction was close to the ones we reported in this study, although the sensorial methods used were different. However, the fining process used in these studies permanently removed the tannins from the wine, thus substantially reducing its nutrient value. The similar approaches to reduce astringency by microencapsulation were reported for polyphenolic-rich plant extract, and the results are of great agreement with ours. When it was microencapsulated by gelatin/gum arabic and gelatin/κ-carrageenan in ice cream, the undesirable astringency sensation of the cinnamon extract was masked, which was confirmed by the sensory trails [50]. In another study, maltodextrin was successfully used as a carrier to mask the astringency of PA for the production of spray-dried cinnamon extract powders [51]. Both studies were conducted in solid food products, whereas our study was the first one carried out in a beverage to the best of our knowledge. All these results suggested that microencapsulation using OSH could be an effective method to mask the astringent taste of PA-rich beverages.

## 4. Conclusions

In this study, the feasibility of using OSH to microencapsulate PA and mask its astringent taste in beverages was demonstrated. When the PA was encapsulated by OSH, it was homogeneously distributed inside the OSH; and the major forces between PA and OSH are hydrogen bonding and electrostatic interaction. Compared to pure PA, such microencapsulation effectively prevented the interaction between PA and the α-amylase in the saliva, leading to a significantly reduced astringent taste (*p* < 0.01). The PA-OSH complex has limited digestion and PA release in the salivary conditions. However, the PAs were mainly released from the complex in the intestinal conditions rather than the salivary and stomach conditions. Such an intestine-specific release would benefit the further adsorption and nutritional utilization of PA. Our findings indicated that the OSH microencapsulation could mask the disliked astringent taste of PA in beverages and maintain its bioavailability at the same time. All these results suggested that the OSH could be used as a new class of food additive to produce polyphenol fortified/enriched beverage products and improve their taste.

Further studies should focus on the influence of OSH on the physicochemical properties of food products, such as rheological behavior, in order to better understand the possible chemical and sensorial alterations. Studies evaluating the performance of the OSH in different food processing methods, such as high-temperature short-time pasteurization and high-pressure processing, are also needed to determine the feasibility of OSH as a new food additive.

## Supplementary Materials

The following are available online at https://www.mdpi.com/2304-8158/9/6/756/s1: Figure S1: The proanthocyanidin (PA) uptake capacity (Γ, mg/g) of the oxidized starch hydrogel (OSH) as a function of the PA concentration. The OSH concentration is 1 g/L for all samples. Figure S2: The ζ-potential of the PA-OSH complex as a function of the PA concentration. The OSH concentration is 1 g/L for all samples.
