# Supplementary material for: Masking the Perceived Astringency of Proanthocyanidins in Beverages Using Oxidized Starch Hydrogel Microencapsulation
Source: Foods. 2020 Jun 8;9(6):756. doi: 10.3390/foods9060756 (PMC7353531; doi:10.3390/foods9060756)
Supplement: Supplementary file 1 [file foods-09-00756-s001.pdf]

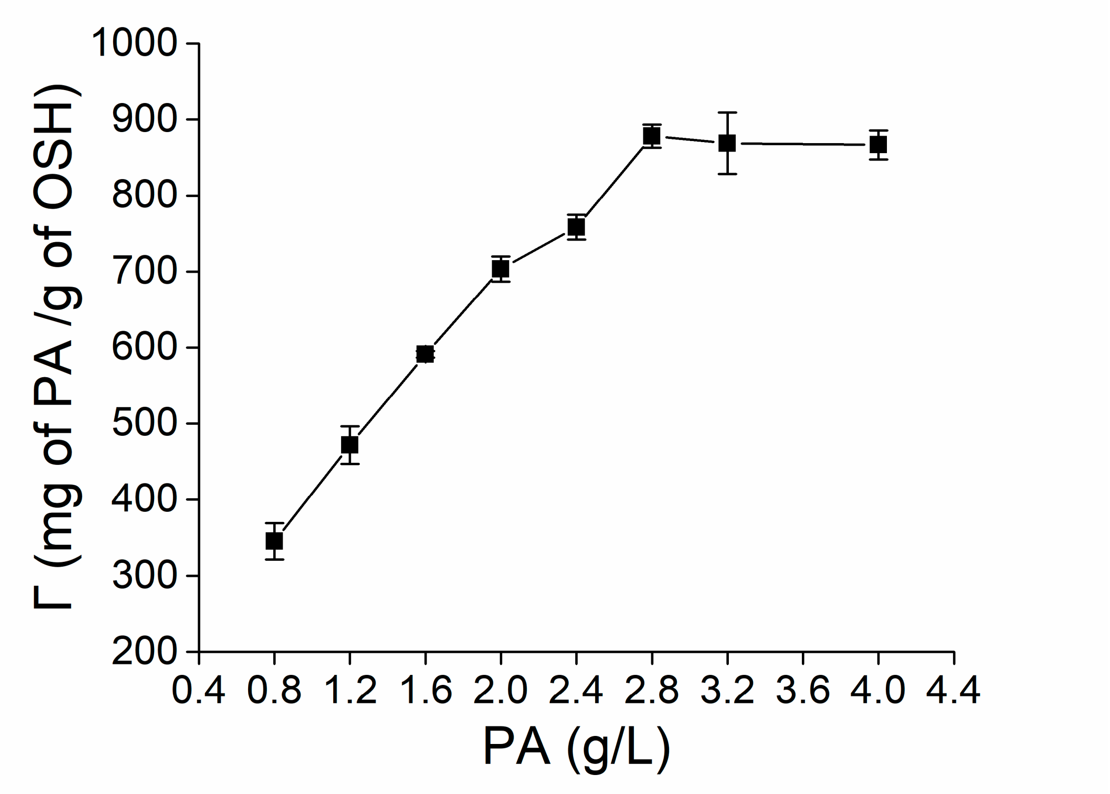

Figure S1. The proanthocyanidin (PA) uptake capacity (Γ, mg/g) of the oxidized starch hydrogel (OSH) as a function of the PA concentration. The OSH concentration is 1 g/L for all samples.

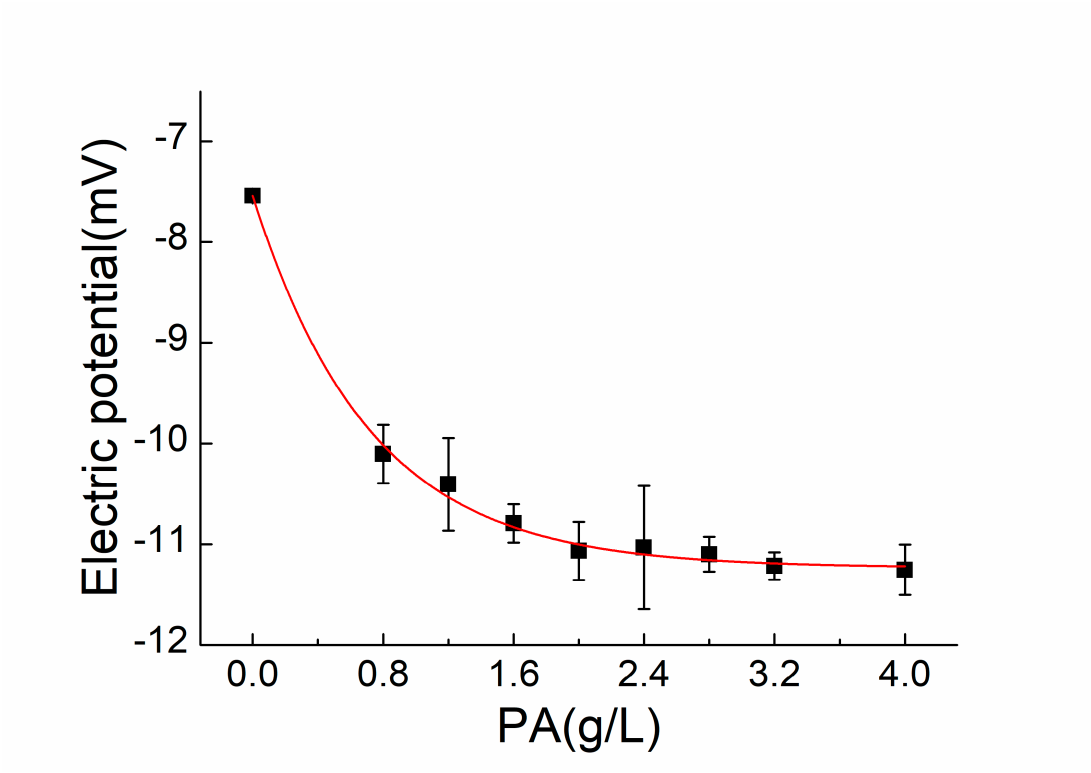

Figure S2. The ζ-potential of the PA-OSH complex as a function of the PA concentration. The OSH concentration is 1 g/L for all samples.
